# Aluminum Cooking Utensils

**Published:** 1915-05

**Authors:** 


**Aluminum Cooking Utensils.** John Gloster of Glasgow University states that the only food stuffs in common use that attack aluminum are: oranges, lemons, Brussels sprouts, tomatoes—to which list, a housewife adds peas. In no case is the amount of aluminum dissolved dangerous.

